# Editorial: Safety Pharmacology – Risk Assessment QT Interval Prolongation and Beyond

**DOI:** 10.3389/fphys.2018.00678

**Published:** 2018-06-08

**Authors:** Eleonora Grandi, Stefano Morotti, Esther Pueyo, Blanca Rodriguez

**Affiliations:** ^1^Department of Pharmacology, University of California, Davis, Davis, CA, United States; ^2^Biomedical Signal Interpretation and Computational Simulation Group, Aragón Institute of Engineering Research, IIS Aragón, University of Zaragoza, Zaragoza, Spain; ^3^Department of Computer Science, University of Oxford, Oxford, United Kingdom

**Keywords:** cardiotoxicity, QT interval prolongation, drug-induced arrhythmia, multi-scale modeling, cardiac electrophysiology

## The need of new paradigms for cardiac safety

The scope of safety pharmacology is to predict whether a drug is likely to cause potentially lethal adverse effects if administered to humans. While safety pharmacology has broadened its interests in recent years to the whole cardiovascular, respiratory, and central nervous systems (and is now extending to other body functions), a major focus since its inception has been assessing drug-induced prolongation in the QT interval—a surrogate biomarker for torsades de pointes (TdP) liability. Because the vast majority of drugs that can cause QT prolongation inhibit hERG channels, current regulatory guidelines concerning cardiac safety recommend that all compounds are evaluated *in vitro* for their hERG inhibitory potency (Redfern et al., 2003) and *in vivo* for their ability to cause QT/QTc interval prolongation (Food and Drug Administration, 2005) in an appropriate animal model and in humans. However, it has now become apparent that QT/QTc prolongation and hERG block are an insufficient proxy for TdP risk. While the current approach based on these markers has been successful in terms of preventing TdP risk, this regulatory paradigm might lead to withdrawal from the drug development pipeline and clinical use of potentially safe drugs. There is therefore a crucial need to develop a more accurate assessment of proarrhythmic potential of drugs. Notably, in 2014 the Comprehensive *in vitro* Proarrhythmia Assay (CiPA) initiative was proposed as a new strategy by expert working groups sponsored by the US Food and Drug Administration (FDA), the Cardiac Safety Research Consortium (CSRC), and the Health and Environmental Science Institute (HESI), and has quickly become a global effort, also involving many industry and academia partners (Sager et al., 2014). CiPA aims at developing and validating a new paradigm for cardiac safety evaluation of new drugs that provides a more accurate and comprehensive mechanistic-based assessment of proarrhythmic (rather than QT prolonging) potential of drugs (Gintant et al., 2016). This involves assessment of (i) high-throughput *in vitro* screening of drug effects on multiple human ion channels, (ii) coupled with *in silico* modeling of human cardiac myocytes to assess integrated electrophysiological responses, and (iii) verification of predicted responses in human induced pluripotent stem cell derived cardiomyocytes (hiPSC-CMs). Safety pharmacology has evolved in recent years to identify and incorporate new technologies for clinical and non-clinical applications, including refinement of *ex vivo* and *in vitro* assays and screens, *in vivo* models, non-invasive clinical modalities, and *in silico* approaches. Here we collected a series of review, perspective, and original research articles that summarize the state of our knowledge and the latest advances in these technologies, and how these might contribute to shaping new and improved cardiac safety guidelines.

## Multiscale modeling for safety pharmacology

**Figure 1:**
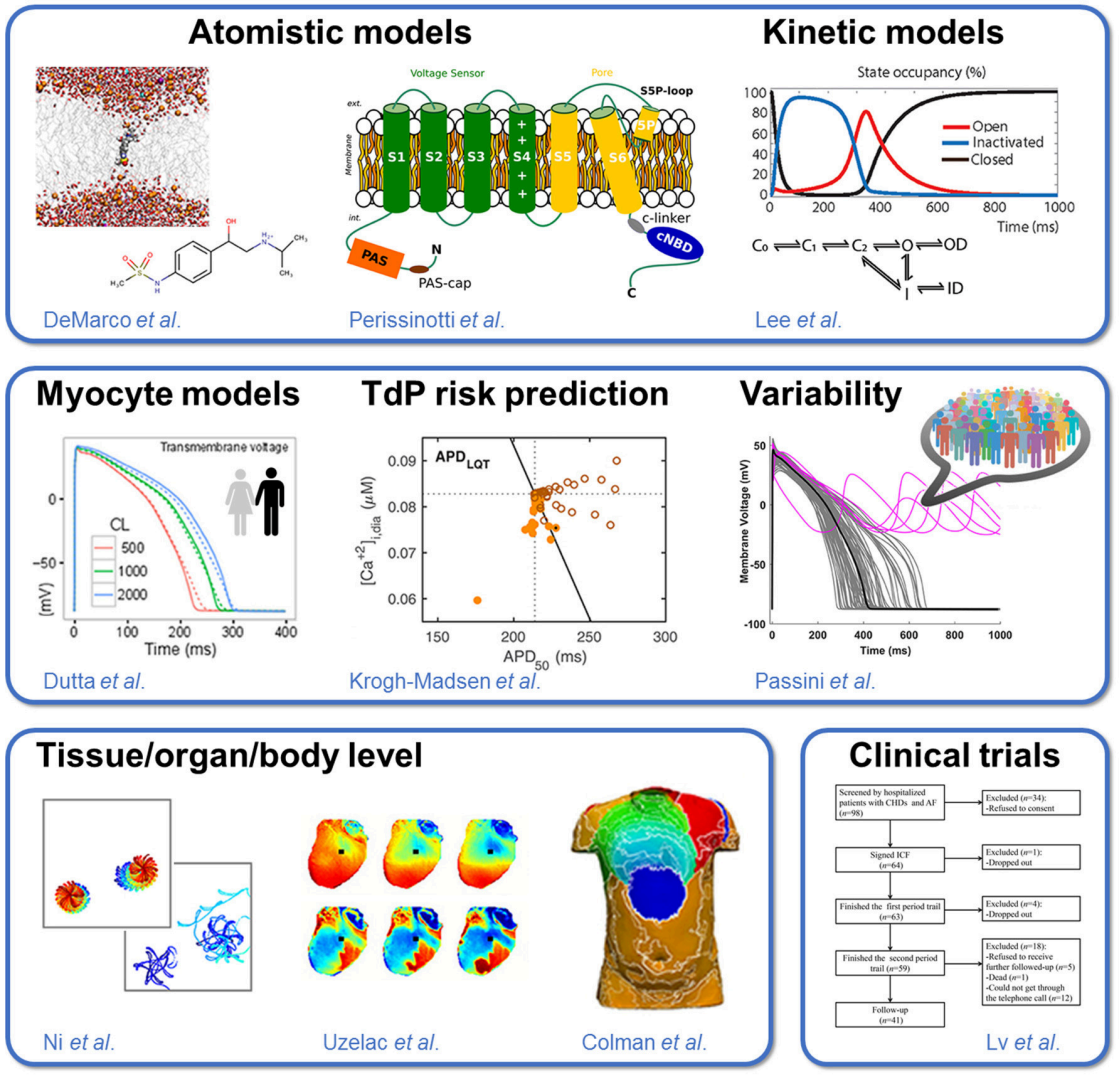
Approaches to predicting drug cardiotoxicity. The Research Topic includes: (1) studies of ion channel structure and function and drug interaction, ranging from atomistic models to kinetic models of whole-cell ion currents **(Top)**; (2) mechanistic models of single cardiac myocyte electrophysiology, development of metrics for quantification of torsadogenic risk, and population-based approaches accounting for cell-to-cell variability **(Middle)**; and (3) tissue/organ/organism level studies spanning tissue-level to whole heart and torso simulations, *ex vivo* recordings in animal preparations, and human clinical trials **(Bottom)**.

There is a wide range of length and time scales covered in this Research Topic, from the atom and ns to the whole organism and month/year (Figure 1 *top left* to *bottom right*), all of which are relevant to safety pharmacology. Structural studies, including modeling of ion channel gating (Perissinotti et al.) and interactions with drugs, and drug partitioning, are critical for drug discovery efforts, and perhaps also a necessary approach for safety considerations. For example, the study by DeMarco et al. utilized all-atom molecular dynamics simulations to show that ionization of drug molecules (specifically Sotalol) can significantly affect their membrane permeability and partitioning kinetics, and should therefore be a consideration in ongoing *in silico* safety pharmacology efforts. Given the complexity of the interaction between drugs and ion channels, the drug binding kinetics, state dependent binding, and temperature dependence could significantly alter drugs' impact on the action potential (AP), even when drugs display similar steady-state block. Lee et al. highlighted some of the challenges involved in modeling of the hERG channel and also discussed limitations and need for improved voltage-clamp protocols to characterize drug-channel interaction in *in vitro* experiments. Ellinwood et al. looked at the consequences of drug binding kinetics and state dependence of K_V_1.5 targeting drugs on atrial electrophysiology, and revealed that ionic remodeling also affects the degree of efficacy and safety of state-specific I_Kur_ inhibitors, by modifying the AP trajectory. These studies highlight the potential need for extraordinary detail in the *in vitro* characterization for accurate *in silico* prediction of (cardiac-region specific, Morotti et al., 2016; Ellinwood et al., 2017; Ellinwood et al.) drug effects on channels and cardiac electrophysiology.

While the ion channel gating and drug-interaction models might require further refinement and increased complexity, significant efforts have been put forward to improve existing cardiomyocyte models and to take advantage of the existence (and convergence) of competing mathematical models to narrow hypotheses or explore alternative hypotheses (Sarkar and Sobie, 2011; Sánchez et al., 2012; Gemmell et al., 2014; Mann et al., 2016; Pueyo et al., 2016a,b; Gong et al., 2017; Muszkiewicz et al., 2017). For example, recent work has shown that, when forced to reproduce the same data, three competing models of human ventricular myocytes (Ten Tusscher and Panfilov, 2006; Grandi et al., 2010; O'Hara et al., 2011) became substantially more similar than they were originally (Mann et al., 2016). Notably, the work by Krogh-Madsen et al. used clinical congenital LQT data (as done by Mann et al., 2016) and physiological constraints on intracellular ionic concentrations to optimize parameters in the O'Hara-Rudy (ORd) human model (O'Hara et al., 2011). This in turn improved the accuracy and robustness of TdP risk prediction (Lancaster and Sobie, 2016), which the authors attributed to the importance of Ca^2+^ dynamics in repolarization and to an improved balance of I_Ks_ vs. I_Kr_ in the new model. A different parameterization of the ORd model by Dutta et al. and Dutta et al. also yielded a better correspondence with drug response data and improved the identification of pro-arrhythmic drugs. The authors developed a new metric qNet, which quantifies the net electronic charge carried by major inward and outward ionic currents during the steady state AP, to separate low-, intermediate-, and high-risk hERG blockers. A follow up study appraised the robustness of qNet as a biomarker for TdP by considering how uncertainty in the model parameters propagates to the phenotype level (Chang et al.). The authors were thus able to identify the conditions under which decisions on risk can be made reliably and objectively. Yet, questions remain regarding the physiological meaning of this new metric, and whether multiple metrics should be utilized that account for a broader range of behaviors and mechanisms. Tixier et al. used an *in silico* model of multi-electrode array electrophysiology and machine learning to identify predictive biomarkers that should be measured to improve classifications of drugs. These investigations add to several previous efforts to build computational frameworks for assessment of TdP risk (Mirams et al., 2011; Kramer et al., 2013; Lancaster and Sobie, 2016). On the other hand, Parikh et al. showed that a simpler classification method based on direct features (ion channel block information) performed with comparable or higher accuracy than existing methods based on simulated metrics. One potential limitation of this approach is that direct feature classifiers might fail identifying the proarrhythmic risk of drugs affecting channels that are not included in the training set, whereas predictive modeling is more likely to yield an accurate classification.

Biophysical modeling can not only provide means for drug classification, but also understanding of the mechanistic underpinning of drug responses, as in the multiscale simulations by Ni et al. and Colman et al. These studies are important reminders that AP duration changes are rarely homogenous (e.g., there exist gradients—transmurally, or from base to apex) and can increase the tissue-level substrates for arrhythmias (Antzelevitch, 2005; Glukhov et al., 2010). Indeed, multiscale *in silico* models can be very powerful tools to investigate the response of candidate antiarrhythmic compounds at the level of the electrocardiogram (ECG). The simulated data may also serve to identify novel ECG-derived biomarkers detecting block of inward and/or outward currents based on ECG features (Vicente et al., 2016). Using Langendorff perfused *ex vivo* rabbit hearts the Fenton group measured and analyzed the complex dynamics of spatially discordant alternans, which provide the substrate for reentrant arrhythmia (Uzelac et al.) The authors noted that current AP models fail to reproduce some key dynamics such as voltage amplitude alternans, smooth development of Ca^2+^ alternans in time, and conduction. Experimental characterization of these dynamics can inform refining of existing models to analyze mechanisms.

## Accounting for patients' condition and inter-subject variability

Clinical risk assessment and trial suggest that patient conditions, i.e., sex (Yang et al., 2017; Vorobyov and Clancy, 2018), age, disease, electrolyte imbalance (Lazzerini et al.), interaction with other drugs (Lv et al.) should all be taken into account in risk assessment (Lane and Tinker)—which is not yet addressed by CiPA efforts. Along the same lines, Wisniowska et al. reviewed the different sources of variability (both intrinsic and extrinsic) that exist in the human population in response to drug action, and emphasized the need of accounting for these aspects in modeling approaches for safety pharmacology. Two studies by the Rodriguez group establish the potential of population-based approaches as very powerful *in silico* tools for safety pharmacology investigations. Passini et al. showed that human *in silico* drug trials using repolarization abnormality quantification as the main metric do better than animal models in detecting drugs with TdP risk. They also show agreement of *in silico* predictions and two established experimental models (rabbit wedge ECGs and hiPSC-CMs). Other statistical methods, e.g., logistic regression, have been previously employed to assess the proarrhythmic risk in a population of computational model variants (Lee et al., 2013; Morotti and Grandi, 2017). Calibrated populations of models of heart cells could generally reproduce experimental drug effects on human tissue for dofetilide, whereas lack of agreement between experiments and simulations for quinidine and verapamil suggest further work is needed to understand the more complex electrophysiological effects of these multichannel blocking drugs (Britton et al.).

## Use of IPSC-CMS for safety pharmacology

Because iPSC-CMs are a readily-obtainable and renewable source of human cardiac myocytes, they are gaining popularity as a platform to screen drugs for toxicity testing. However, given the iPSC immature phenotype, and phenotypic differences across iPSC-CM cell lines (Lei et al.), it remains unclear how well drug tests performed in iPSC-CMs will recapitulate the effects observed in adult human cardiomyocytes and hearts. Koivumaki et al. developed a computational model of the iPSC-CMs that recapitulates the cells' immature phenotype, and explore differences in ionic behavior underlying the AP in paced vs. spontaneous modes, phenotypic variability in iPSC-CMs, and iPSC-CM model's ability to recapitulate physiological properties of adult cells. Recently, statistical methods have also been established to provide accurate predictions of adult myocyte drug responses from iPSC-CM simulations (Gong and Sobie, 2018). iPSC-CM utilization in drug discovery and safety investigations is reviewed by Ortega et al. An important advancement in the technological approach of improving the utility of iPSC-CMs for safety pharmacology is the augmentation of I_K1_ using dynamic clamp. Plagued by low-throughput, Goversen et al. have moved toward demonstrating that such dynamic clamp can be performed in a high throughput manner. Bjork et al. reported that the expression of optogenetic tools in iPSC-CMs did not significantly affect the baseline electrophysiological properties of these cells, thus allowing electrophysiological assessments comparable to conventional patch clamp studies. Nevertheless, adult human ventricular cardiomyocytes (Nguyen et al.) and trabeculae (Qu et al.) might still be a more reliable model to test the cardiotoxic risk associated with novel drugs, with some advantages over animal and iPSC models.

## Conclusions and future directions

There is a growing body of work supporting the integration of new and established computational and experimental approaches to understanding and predicting the risk of TdP. While mechanistic systems modeling is mature in the cardiac arrhythmia field, use of similar approaches can improve understanding and prediction of cardiotoxicity caused by other drugs, e.g., cancer therapeutics (Shim et al.). Given the focus on TdP and QT interval, however, the deleterious effects of drugs on cardiac function are evaluated only in terms of changes in electrophysiological properties. Future work should therefore extend the current paradigm to include other major cellular functions (such as contraction, energetics, and cell death, i.e., via apoptosis), which dysregulation can severely impact cardiac performance. In addition to cardiotoxicity, safety pharmacology aims to determine the potential undesirable pharmacodynamic effects of a drug on the central nervous, vascular and respiratory systems (Pugsley et al., 2008). Thus, extension of the described approaches to these systems seems desirable, and might contribute to further advancement of these key areas of biomedical research.

## Author contributions

EG and SM wrote the editorial. EP and BR provided comments and edits.

### Conflict of interest statement

The authors declare that the research was conducted in the absence of any commercial or financial relationships that could be construed as a potential conflict of interest.
